# DEGLUTO: initial validity evidence of a clinical swallowing assessment tool for adults

**DOI:** 10.1590/2317-1782/e20250153en

**Published:** 2026-05-08

**Authors:** Jadson Stheferson Dutra Alves, Leandro de Araújo Pernambuco, Hipólito Virgilio Magalhães

**Affiliations:** 1 Departamento de Fonoaudiologia, Universidade Federal do Rio Grande do Norte – UFRN - Natal (RN), Brasil.; 2 Departamento de Fonoaudiologia, Universidade Federal de Pernambuco – UFPE - Recife (PE), Brasil.

**Keywords:** Swallowing, Swallowing Disorders, Assessment, Validation Study, Speech-Language Pathology

## Abstract

**Purpose:**

To develop and verify content validity evidence of DEGLUTO, a clinical swallowing assessment instrument for adults.

**Methods:**

A cross-sectional methodological study. The first phase validated the content using the Delphi method with 13 judges, analyzing 28 items and using CVI, CVC, and Kappa for agreement. The second phase evaluated response processes by applying the instrument to 210 adult and elderly patients by ten speech therapists, analyzing application time, acceptability, and inter-rater agreement.

**Results:**

In content validation, seven items were removed and twenty-one reformulated, resulting in 21 items with a mean CVI > 0.90 and substantial to almost perfect agreement. In response processes, a mean application time of 15 minutes, good acceptability, and high agreement on most items were observed. One item was excluded, consolidating the final version with 20 items.

**Conclusion:**

DEGLUTO presented robust initial validity evidence, demonstrating clinical applicability, clarity, and theoretical adequacy, qualifying it as a promising tool for the assessment of oropharyngeal dysphagia in clinical practice.

## INTRODUCTION

Oropharyngeal dysphagia (OD) is a clinical condition characterized by functional impairment of swallowing, making it difficult or impossible to ingest food and liquids safely and efficiently, with a negative impact on the quality of life^(1)^. It is associated with potentially serious complications, such as malnutrition, dehydration, aspiration pneumonia, and an increased mortality rate, which reinforces the need for a systematic and accurate assessment for its proper diagnosis and management^(2)^.

OD is a common condition that can affect a wide variety of clinical populations, with an estimated prevalence of up to 33.7% in the general population, being even higher in hospitalized individuals (41%) and in people diagnosed with stroke (55.2%)^(3)^. Among older people, the occurrence ranges from 18.3% in community-dwelling individuals to 46.9% in residents of long-term care facilities^(4)^. In other neurological conditions, such as Parkinson's disease, it can affect up to 80% of patients^(5)^. Moreover, dysphagia affects approximately 50% of people with head and neck cancer, especially after interventions such as surgery and radiotherapy^(6)^.

Thus, the clinical assessment of swallowing is an essential step for the early diagnosis and proper management of dysphagia^(2)^. Using instruments submitted to the validation process strengthens evidence-based practice, providing greater diagnostic accuracy and contributing to the definition of safer, more effective, individualized therapeutic approaches^(4)^.

However, for these instruments to reliably fulfill their clinical role, they must be developed and evaluated based on robust validity criteria, which refers to the adequacy of the inferences made from the results of an instrument, being a central attribute for its clinical usefulness^(7)^. According to ISPOR (The Professional Society for Health Economics and Outcomes Research), the construction of validity should consider the target construct, the population of interest, the context of application, and the consistency of interpretations resulting from instrument use^(7)^.

Hence, content validity is one of the fundamental steps in the development of clinical instruments. It concerns the degree to which the items are representative of the target construct and appropriate to the context in which the instrument will be applied^(7)^. According to ISPOR best practices, this analysis should be conducted by qualified experts who assess the clarity, relevance, and comprehensiveness of the items, ensuring their adherence to the proposed conceptual definition and intended use^(7-9)^.

ISPOR also highlights the importance of understanding how respondents interact with the instrument, ensuring that the items are interpreted consistently with the target construct^(9)^. This step, related to the analysis of response processes, contributes to strengthening the validity of the instrument, especially regarding measures based on clinical assessment, such as ClinROs. The observation and interpretation of these processes provide essential qualitative evidence to ensure that the instrument produces consistent and useful results in clinical practice^(7,9)^.

Validated instruments are essential for reliable diagnoses and effective therapeutic approaches in the management of dysphagia. To this end, they must be constructed with methodological rigor and clinical applicability. According to ISPOR guidelines, the validity of an instrument considers both the representativeness of the items and how they are interpreted by respondents. Thus, this study aimed to obtain initial validity evidence based on the content and response processes of DEGLUTO, according to methodological recommendations for ClinRO instruments^(7-9)^.

## METHODS

### Study design

This methodological study aimed to obtain initial validity evidence based on the content and response processes of DEGLUTO: Clinical Assessment Instrument of Swallowing in Adults (Appendix A). The objective is to support, in future stages, the continuation of the instrument validation process in a population of adults and older adults, according to the good methodological practices recommended by ISPOR^(7-9)^.

The project was submitted to the Research Ethics Committee of the Onofre Lopes University Hospital of the Federal University of Rio Grande do Norte (HUOL/UFRN), in accordance with Resolution 466/2012 of the Brazilian National Health Council, and approved under opinion number 6.934.805. The collaborating speech-language-hearing (SLH) pathologists who agreed to participate in the research and the patients to whom the instrument was applied signed an informed consent form.

### Obtaining content-based validity evidence

This study used the Delphi technique, a structured method that aims to reach consensus among experts through successive rounds of evaluation, carried out anonymously and independently. This procedure favors informed decisions and reduces the influence of group biases^(10)^. In each round, participants received a statistical summary of previous responses, allowing them to review and refine their assessments.

The Brazilian version of the Northwestern Dysphagia Patient Check Sheet (NDPCS), previously translated and cross-culturally adapted, was used as the basis for developing the initial set of 28 DEGLUTO items. These items were submitted to the evaluation of expert judges regarding clarity, relevance, and adequacy to the proposed construct (OD identification).

### Research location and reference period

The phase of gathering content validity evidence was conducted remotely, contacting expert judges via email over three evaluation rounds. Data were collected and analyzed between July and November 2024, as the experts actively participated in the instrument review and refinement process, in accordance with the methodological principles previously described.

### Population

The sample consisted of expert judges in OD, selected by convenience based on their clinical, teaching, and/or research experience in the field, with a minimum of 5 years of experience. They were selected in a meeting between the lead researchers, considering criteria of professional recognition and expertise in the subject. Then, a total of 23 experts were formally invited via email to compose the evaluation panel.

### Data collection procedures and instruments

Participants were sent an invitation letter via email with guidelines about the research, an informed consent form, and a preliminary version of the instrument to be evaluated.

The evaluation was conducted using a structured electronic form based on a Likert-type scale: "strongly agree," "agree," "neutral," "disagree," and "strongly disagree." Each DEGLUTO item was analyzed for its relevance, timeliness, clarity, and comprehensiveness. In cases of disagreement, the judges were to justify their responses, suggest changes, and include comments or propose new items deemed relevant.

The process took place in three successive rounds. At the end of each stage, the responses were systematically compiled and discussed in meetings among the researchers, based on scientific literature. These meetings carefully appraised the judges' contributions to maintain, modify, or exclude items, in order to ensure conceptual consistency and the adequacy of the content to the instrument's objective.

### Data analysis

The data were organized in a spreadsheet in .xlsx format and analyzed using Microsoft Excel 2024, which was also used to construct descriptive tables.

The analysis of the content validity of DEGLUTO was based on three complementary metrics: the Kappa coefficient, the content validity index (CVI), and the content validity coefficient (CVC). A minimum agreement of 70% among the experts was adopted as the criterion for maintaining or excluding items.

The Kappa coefficient was applied to verify interrater consistency regarding decisions about the items. The interpretation followed the classification proposed by Landis and Koch^(11)^: values < 0 indicate a lack of agreement; 0.00–0.19, weak agreement; 0.20–0.39, fair; 0.40–0.59, moderate; 0.60–0.79, substantial; and 0.80–1.00, near-perfect agreement.

CVI was calculated according to Pasquali's^(12)^ criteria, being divided into two dimensions: CVI per item (I-CVI), corresponding to the proportion of positive responses ("agree" or "strongly agree"), and the general CVI (G-CVI), obtained by averaging the I-CVI per domain. Values ​​greater than 0.78 for I-CVI and 0.90 for G-CVI were considered acceptable.

The CVC was determined based on Hernández- Nieto's^(13)^, proposal in five successive steps. First, the initial CVC (CVCi) was obtained from the average of the evaluators' scores (scale of 1 to 5). This value was corrected by an estimated error (Pei), resulting in the corrected CVC (CVCc) for each item. The total CVC (CVCt), in turn, corresponded to the average of the CVCc of each dimension of the instrument. Values ​​above 0.8013 were considered acceptable.

The researchers analyzed the items with indices below the established parameters and all qualitative suggestions presented by the judges. They made the necessary modifications after discussion in consensus meetings, resulting in a revised preliminary version of the instrument.

### Obtaining validity evidence based on response processes

This stage was conducted with the participation of 10 collaborating SLH pathologists, who applied DEGLUTO in different clinical contexts. After the application, the professionals were invited to evaluate aspects related to the items’ clarity and comprehensiveness, the patients' comprehension as they used the instrument, and its general clinical applicability.

The researchers also considered the average application time and acceptability of the instrument among the evaluators. The analysis of these elements made it possible to identify how the respondents interacted with DEGLUTO, ensuring that the items were interpreted according to the intended construct.

### Research location and reference period

The research took place in HUOL/UFRN’s OD outpatient clinic and in the workplace of the collaborating SLH pathologists. The application scenarios encompassed two high-complexity public hospitals (a general university hospital and a referral hospital in neurology, trauma, and burns), a large private hospital, and two public hospital outpatient clinics specialized in the care of OD patients.

The applications were carried out in intensive care units (ICUs), clinical and surgical wards, dysphagia, neurology, and head and neck outpatient clinics, and emergency rooms. This broad geographical and typological distribution of research environments aimed to test the applicability of the instrument in different care realities, from acute care to outpatient follow-up. All study stages followed the ethical and methodological criteria previously approved by the Research Ethics Committee. Data were collected between November 2024 and February 2025.

### Population

The study sample consisted of 210 adult and older patients (mean age 73.67 years, 58.10% > 70 years, mostly female), referred for SLH evaluation due to complaints of OD. Selected by convenience, it included a wide range of clinical conditions associated with dysphagia, such as frail older adults, neurological pathologies (stroke, neurodegenerative diseases), complications from prolonged hospitalization, post-intubation, tracheostomy, use of alternative feeding routes, decompensated respiratory diseases, head and neck cancer/burns, and trauma. This diversity aimed to ensure the representativeness and broad applicability of DEGLUTO in different contexts, following the recommended 10 patients per item.

DEGLUTO was applied by 10 collaborating SLH pathologists, all with more than 5 years of training and experience in OD management. Four of them held a master's degree, one a doctorate, one had completed a multi-professional residency, one was a specialist, and three worked exclusively in clinical care. Seven of them applied the instrument in hospitals, and three worked in outpatient settings. This professional and institutional diversity allowed for a comprehensive analysis of the clinical applicability of DEGLUTO in different care scenarios.

### Data collection procedures and instruments

The process involved the application of DEGLUTO in various clinical contexts by participating SLH pathologists. To ensure uniformity, all professionals received standardized guidelines in an in-person meeting with the researcher, when the instrument's calibration manual was detailed and delivered (Appendix B).

The SLH pathologists applied DEGLUTO to patients with complaints of OD, evaluating aspects such as item clarity and comprehensiveness, patient comprehension, clinical applicability of the instrument, average application time, and overall acceptability.

After finishing the applications, each professional received an individual electronic form, sent by email, in which they recorded their perceptions about the adequacy of the instrument, possible difficulties encountered, and suggestions for improvement. This information was systematically analyzed to identify aspects to be adjusted in the instrument, improving accuracy and practical usefulness.

Each DEGLUTO item was evaluated using two Likert-type scales, both with five response options: “strongly agree,” “agree,” “neutral,” “disagree,” and “strongly disagree.” The first scale referred to the item’s adequacy according to the examiner’s perception; the second, to its clarity and the patient’s comprehension. In addition to the scales, the forms contained open fields for qualitative comments, allowing for a more in-depth analysis of the experience of using the instrument.

### Data analysis

Content validity and validity based on response processes underwent standardized analyses, using the same methodological criteria and statistical tools. CVI, CVC, and the Kappa coefficient were applied, allowing us to measure interrater agreement and item representativeness and clarity in the clinical context.

This methodological standardization ensured greater consistency in the analyses and enabled comparability between the evidence obtained in the different stages of the validation process. The use of multiple statistical indicators also contributed to a more robust assessment of the instrument's quality, aligning with the recommendations of international best practices for the validation of clinical instruments.

## RESULTS

### Obtaining content-based validity evidence

Thirteen of the 23 judges invited to participate in this stage accepted the invitation and responded to the first round of the questionnaire. Seven of them had predominantly academic backgrounds (university professors and researchers with more than 5 years of experience in dysphagia and relevant publications in the field), and six had greater experience in clinical and care practice (SLH pathologists with more than 5 years of experience in high-complexity hospitals and dysphagia clinics). The diversity of profiles among the judges aimed to ensure a comprehensive evaluation of both the theoretical perspective and the practical applicability of the instrument.

### Round I

The initial version of DEGLUTO had 28 items. After the first round of evaluation, five questions were removed because their concordance rate among the judges was below 70%, with low clinical relevance and little direct relationship with the construct being evaluated. Moreover, 21 items were reformulated, with adjustments to the wording and structure to improve clarity, reduce ambiguities, and ensure more accurate application. These modifications aimed to improve the evaluators' comprehension, ensuring that the items were interpreted uniformly and aligned with the needs of clinical practice. The following items were excluded: 1. History of recurrent pneumonia, 7. Attention/interaction skills, 9. Awareness of secretions, 14. Ability to follow directions, and 19. Pharyngeal contraction in the gag reflex. The overall agreement indices in this round were Kappa = 0.70, CVI = 0.90, and CVC = 0.90, indicating substantial agreement among the judges and the adequacy of the remaining items.

### Round II

In the second round, 11 of the 13 judges responded to the questionnaire; two specialists were absent, both with clinical experience. Thus, the evaluation group consisted of seven academic judges and four clinical judges.

The revised instrument had 23 remaining questions. Three of them had a concordance rate of less than 70%, namely: 2. Presents frequent episodes of fever, 6. Uncooperative, and 7. Lack of awareness of the swallowing problem. After a literature review and consensus among the lead researchers, they removed questions 2. Presents frequent episodes of fever and 7. Lack of awareness of the swallowing problem, and reformulated question 6. Uncooperative as "inappropriate behavior during meals". The remaining 21 questions were maintained, achieving an average concordance of 85%. The overall indices in this round were Kappa = 0.75, CVI = 0.92, and CVC = 0.91, demonstrating an improvement in the consistency of the instrument.

### Round III

In the third round, nine of the 11 judges who participated in the previous phase responded to the questionnaire. One academic specialist and one clinician did not participate in this stage, consolidating the final panel with six academics and three assistants.

The exclusive focus was on the reassessment of the reformulated question 6. Inappropriate behavior during meals, which obtained 70% agreement among the judges. The agreement indices for this item were Kappa = 0.22, CVI = 0.78, and CVC = 0.82, indicating a fair agreement.

This stage concluded the process of updating the instrument according to the judges, following the principles of the Delphi method. It ensured that the modifications were consistent with the objective of the study and appropriate to the construct evaluated by the instrument.

The results obtained during the instrument evaluation and update process are presented in Table 1, organized to highlight the main changes made throughout the process and the agreement indices obtained at the end of the three rounds.

**Table 1:** Obtaining validity evidence based on the content of DEGLUTO: Clinical Assessment Instrument of Swallowing in Adults

**No.**	**Original Item**	**Judges’ Responses**	**Researchers’ Update**	**Final Agreement Index (%)**
1	History of recurrent pneumonia	Redundant item; they suggest combining it with another	Item removed	69%
2	Frequent episodes of fever	Little direct correlation with dysphagia; they suggest removing it	Item removed	25%
3	Problem with aspiration pneumonia	Full agreement regarding the relevance of the item	History of aspiration pneumonia or recurrent pneumonia	100%
4	Long-term intubation (1 or more weeks) or tracheostomy (6 or more months)	Intubation and tracheostomy time needs to be better defined	Prolonged intubation (48 or more hours) and/or current or previous tracheostomy	100%
5	Alertness	Vague term; suggestions include specifying absentmindedness or fluctuation	Absentmindedness or fluctuating alertness	100%
6	Cooperation	Suggestion: detailing behavior during meals	Inappropriate behavior during meals	70%
7	Attention/interaction skills	Subjective criterion; not relevant for dysphagia	Item removed	67%
8	Awareness of the swallowing problem	Limited clinical applicability; removal is suggested.	Item removed	62.50%
9	Awareness of secretions	Low clinical reliability; lack of consensus on relevance.	Item removed	56.25%
10	Ability to handle secretions	They agree that the wording of the item should be clearer	Difficulty in managing oropharyngeal secretions	90%
11	Postural control	Relevant item; need to specify	Inadequate postural control: trunk and neck instability	100%
12	Fatigability	They acknowledge its relevance but recommend greater objectivity.	Signs of fatigability	90%
13	Oral, pharyngeal, and laryngeal anatomy/physiology	Need for reformulation to improve applicability	Possible alterations in the anatomy and physiology of the mouth, pharynx, or larynx	90%
14	Ability to follow directions	This item is not essential for dysphagia assessment	Item removed	56%
15	Dysarthria	They suggest maintaining it as a relevant criterion for dysphagia	Presence of dysarthria	100%
16	Orofacial muscle tone	They agree on its importance but recommend adjusting the terminology.	Impaired orofacial muscle tone	90%
17	Oral apraxia	Relevant item; further details needed	Presence of oral apraxia	81%
18	Orofacial sensitivity	Relevant item, description needs adjustments	Changes in orofacial sensitivity	100%
19	Pharyngeal contraction in the gag reflex	Low correlation with dysphagia; removal recommended.	Item removed	69%
20	Swallowing saliva	It is necessary to detail the evaluation criteria.	Accumulation of saliva in the oral cavity	100%
21	Voluntary cough and throat clearing	Relevant item, but needs reformulation for better clarity	Absent or inefficient voluntary cough and throat clearing.	100%
22	Swallowing apraxia	They suggest refining the terminology for greater accuracy	Difficulty swallowing on command	90%
23	Oral residue	They agree on the relevance but recommend greater clarity.	Presence of oral residue after swallowing.	100%
24	Cough and throat clearing	Important for dysphagia assessment; maintain	Presence of swallowing-related cough and throat clearing	100%
25	Delayed pharyngeal swallowing	They suggest further details about the delayed pharyngeal phase	Difficulty initiating the pharyngeal phase	81%
26	Reduction in laryngeal elevation	Adjustments needed for greater specificity in the description.	Reduction in hyolaryngeal movement	100%
27	Wet voice	Agreement with clinical relevance; wording adjustments	Change in voice quality after swallowing	100%
28	Multiple swallows	They suggest specifying the number	Multiple swallows per bolus (3 or more)	100%

After defining the 21 preliminary questions of the DEGLUTO test, a calibration manual was developed for the administrators to ensure standardized instrument use. This manual contains detailed guidelines on the interpretation of each item, scoring criteria, and guidelines to minimize variations between evaluators, ensuring more reliable data collection. Standardizing the application is essential to reduce discrepancies in assessment and improve the accuracy of results (Appendix B).

### Obtaining validity evidence based on response processes

The preliminary version of DEGLUTO was applied to a total of 210 patients by 10 collaborating SLH pathologists.

The instrument application revealed that the response profiles varied consistently with the etiology of dysphagia in the different clinical populations studied. Patients with neurological conditions, such as stroke and neurodegenerative diseases, presented a greater number of suggestive items in all five categories of the instrument. The most prominent findings in this group included abnormal results in the oral motor test, such as dysarthria and impaired muscle tone, and signs of risk during swallowing, such as coughing and impaired vocal quality.

In contrast, groups whose dysphagia was secondary to a systemic impairment, such as frail older adults, post-intubation patients, tracheostomy patients, or those with prolonged hospitalization, presented a pattern characterized predominantly by postural instability, fatigability, and inefficient voluntary cough, reflecting generalized muscle weakness and physical deconditioning.

Moreover, the profile of patients with head and neck cancer was more concentrated in items related to anatomical and physiological impairments, resulting in specific functional deficits, such as oral residue and reduced hyolaryngeal movement. As expected, individuals with a history of aspiration pneumonia or with alternative feeding routes scored positively on the medical history items and obtained high scores in the other domains, corroborating the severity that justified the initial intervention.

The results demonstrated high agreement for most items of the instrument, with Kappa values ​​greater than 0.80, indicating substantial or near-perfect agreement between the applicators. The CVI and CVC also had values ​​above 0.90 for 20 of the 21 items, reinforcing the suitability of the instrument in clinical practice.

Only item 4, "Inappropriate behavior during meals," presented results below the established criteria for acceptance, with Kappa = 0.14, CVI = 0.75, and CVC = 0.82. These values ​​indicate that the evaluators’ interpretation was little standardized, motivating its exclusion after critical analysis and literature review. Interrater agreement is described in Table 2.

**Table 2:** Interrater agreement per question

**Dimension**	**Item**	**Kappa**	**CVI**	**CVC**
**Medical history**	1. History of aspiration pneumonia or recurrent pneumonia.	1.00	1.00	1.00
	2. Prolonged intubation (48 or more hours) or current or previous tracheostomy.	1.00	1.00	0.95
**Behavioral aspects**	3. Absentmindedness or fluctuating alertness.	1.00	0.71	0.92
	4. Inappropriate behavior during meals.	0.14	0.75	0.82
	5. Difficulty in managing oropharyngeal secretions.	1.00	1.00	0.90
**Gross motor function**	6. Inadequate postural control: trunk and neck instability.	1.00	1.00	0.90
	7. Signs of fatigability.	1.00	1.00	0.92
**Oral motor test**	8. Possible alterations in the anatomy and physiology of the mouth, pharynx, and larynx.	1.00	1.00	0.87
	9. Presence of dysarthria.	1.00	1.00	0.92
	10. Impaired orofacial muscle tone.	1.00	1.00	0.95
	11. Presence of oral apraxia.	1.00	1.00	0.92
	12. Changes in orofacial sensitivity.	1.00	1.00	0.92
	13. Accumulation of saliva in the oral cavity.	1.00	1.00	0.97
	14. Absent or inefficient voluntary cough and throat clearing.	1.00	1.00	0.95
**Observation during swallowing tests**	15. Difficulty swallowing on command.	1.00	1.00	0.95
	16. Presence of oral residue after swallowing.	1.00	1.00	0.95
	17. Presence of swallowing-related cough and throat clearing.	1.00	1.00	0.97
	18. Difficulty initiating the pharyngeal phase.	1.00	1.00	0.95
	19. Reduction in hyolaryngeal movement.	1.00	1.00	0.95
	20. Change in voice quality after swallowing.	1.00	1.00	0.95
	21. Multiple swallows per bolus (3 or more).	1.00	1.00	0.92
	General	0.96	0.99	0.93

**Caption:** CVI = content validity index; CVC = content validity coefficient. Source: Developed by the authors

The measurement of the application time revealed that DEGLUTO administration took 12 to 20 minutes, with an average of 15 minutes per patient. The acceptability of DEGLUTO was evaluated using an electronic questionnaire. The analysis of the responses indicated good acceptance, highlighting the objectivity, ease of application, and suitability of the instrument to different clinical contexts.

## DISCUSSION

Obtaining validity evidence based on content and response processes is fundamental to ensuring that an instrument accurately reflects the construct being assessed and has clinical applicability. This process follows the international best practices of ISPOR, which guide the development of valid, standardized, and practice-relevant instruments^(7-9)^. The Delphi method allowed for a thorough analysis of DEGLUTO, resulting in adjustments that improved its clarity and clinical relevance.

The exclusion of the item "history of recurrent pneumonia" was based on the literature, which points to the complexity of the relationship between pneumonia and dysphagia. Although aspiration is a risk factor, recurrent pneumonia involves multiple variables, such as immunity, oral hygiene, functional dependence, and comorbidities^(14)^. Its inclusion, despite appearing to increase the scope of the instrument, could generate misinterpretations and reduce the specificity of DEGLUTO, as it would not necessarily indicate a swallowing problem per se, but a multifactorial consequence.

The question “attention/interaction skills” was excluded because it is a subjective and nonspecific criterion for assessing dysphagia. Although cognitive deficits can interfere with feeding, they do not always determine the presence of swallowing disorders. Studies indicate that attentional capacity can influence eating behavior, but its direct relationship with swallowing effectiveness is not conclusive^(15)^.

The item “awareness of secretions” was removed from the instrument due to its low clinical reliability, since the patient's perception of the presence and management of oral and pharyngeal secretions can be highly variable. This variability is even more pronounced in individuals with neurological impairment, who frequently present with reduced laryngeal and pharyngeal sensitivity, impacting their ability to detect and eliminate secretions accumulated in the upper airway^(14)^.

The item “ability to follow directions” was excluded because cognitive deficits do not necessarily directly impact the mechanics of swallowing. Patients may present with comprehension difficulties without significant impairment in the oral or pharyngeal phase of swallowing, making this issue less relevant within the scope of the instrument^(15)^.

The item “pharyngeal contraction in the gag reflex” was excluded because it was not directly correlated with swallowing effectiveness. Studies show that the presence or absence of this reflex does not predict the risk of aspiration and is not a reliable marker for OD^(16)^. Healthy individuals may not present the reflex without functional impairment, and its absence is also not associated with higher rates of aspiration in neurological patients^(16)^.

The item “presents frequent episodes of fever” was removed due to its low specificity as an isolated indicator of dysphagia, since fever is a nonspecific clinical sign, frequently associated with various infectious causes unrelated to swallowing, such as urinary tract infections, viral infections, and sepsis. Although it can occur in cases of aspiration pneumonia, it is not always present, which limits its usefulness as a clinical marker and can lead to misinterpretations^(14)^. Its inclusion could generate diagnostic biases and lead to misinterpretations.

The item “lack of awareness of the swallowing problem” was excluded due to the low reliability of self-perception as a diagnostic criterion. In neurological conditions, patients often underestimate their swallowing difficulties, which can result in a false sense of security and a higher risk of aspiration^(4)^. Studies with FEES and VFSS show that many do not recognize their own dysphagia, especially in the presence of silent aspiration, which compromises diagnostic accuracy when relying on subjective perception^(5)^. By removing this item, DEGLUTO focuses on objective and observable signs of dysphagia, ensuring more accurate and standardized clinical assessment.

The item “uncooperative” was reformulated to “inappropriate behavior during meals” to broaden clinical applicability and reduce bias. The new wording focuses on observable behaviors (refusal, distraction, resistance to handling), which impact aspiration risk, feeding effectiveness, and adherence, especially in neurological and psychiatric populations. In advanced dementia, apathy and resistance to assisted feeding increase nutritional and respiratory risks^(5,14)^. The reformulation aligns the assessment with clinical practice and promotes effective interventions.

The DEGLUTO content validation process resulted in 21 preliminary items, based on rigorous methodological criteria that ensured clarity, relevance, and clinical applicability. Exclusions and reformulations were based on the literature and the experience of experts, prioritizing objective clinical markers directly related to OD. This refinement contributed to a more standardized and assertive assessment, favoring the early identification of swallowing disorders and greater safety in clinical decisions in hospital and outpatient settings.

After the content validation phase, the analysis of the response processes was fundamental to evaluating the clarity, applicability, and comprehension of the items by professionals and patients, ensuring the consistent use of the instrument in clinical practice^(17)^. The application of DEGLUTO by experienced SLH pathologists in different care scenarios demonstrated its functionality and high agreement among evaluators in most items, reinforcing its reliability, standardization, and clinical applicability.

The application of DEGLUTO demonstrated that the patients' response profiles varied consistently with the etiology of dysphagia, which corroborates the existing literature on the complexity of these conditions^(1,18)^. Neurological patients presented more suggestive findings in all categories, highlighting oral motor deficits (dysarthria and impaired muscle tone) and risks during swallowing (cough and vocal change), aligned with what is described about the neurological impact on swallowing^(5)^.

In contrast, frail older patients and post-intubation, tracheostomy, or prolonged hospitalization patients exhibited patterns of postural instability, fatigability, and cough inefficiency, reflecting generalized muscle weakness^(4)^. In patients with head and neck cancer, the identification of anatomical and physiological changes predominated, such as oral residue and reduced hyolaryngeal movement, justified by structural impairment^(6)^. Finally, the record of a history of aspiration pneumonia and the use of alternative feeding routes validated the instrument's ability to identify the pre-existing severity of dysphagia^(19)^.

A critical point identified was the low agreement in the item "Inappropriate behavior during meals," with Kappa = 0.14, CVI = 0.75, and CVC = 0.82, below the acceptance criteria. The applicators’ inconsistent interpretation compromised its clinical usefulness, possibly due to the subjectivity involved in behavioral aspects, influenced by factors such as cognition, motivation, and emotional state^(14)^. Its exclusion aimed to preserve the objectivity and reproducibility of the instrument, avoiding bias in the evaluation and strengthening the diagnostic accuracy of DEGLUTO.

The analysis of the DEGLUTO application time (average of 15 minutes, ranging from 12 to 20) was considered adequate for clinical practice, aligned with the literature on the feasibility of instruments in time-constrained contexts^(20)^. The variation was influenced by the patient's severity, the applicator's experience, and familiarity with the instrument, according to clinical applicability studies in dysphagia^(20)^. Experienced professionals tend to reduce the time, while more compromised patients require more.

The acceptability of DEGLUTO by professionals was positive, who described it as clear, systematized, and easy to apply. The literature highlights that instruments with a scientific basis and objective structure favor clinical adherence by minimizing ambiguities and interpretative variations^(20)^.

The analysis of the response processes showed that DEGLUTO is feasible, applicable, and reliable for clinical practice. The high interrater agreement, the appropriate application time, and the positive reports from professionals reinforce its usefulness in swallowing assessment, contributing to more accurate diagnoses and standardized clinical practices.

Despite the positive results, some limitations should be considered. Judge participation decreased throughout the rounds of the Delphi method in the content validation stage. However, although the number of participants varied between rounds, decisions were made based on rigorous statistical agreement criteria, which ensured the validity of the content even with panel fluctuations. Furthermore, item reformulations and exclusions were always based on in-depth discussion among the researchers and on current scientific literature, minimizing the impact of lower adherence and ensuring the conceptual consistency of DEGLUTO.

Although the sample in the response process stage was selected by convenience and in specific clinical contexts, the process followed internationally recognized guidelines by ISPOR. The diversity of DEGLUTO application scenarios in this study is a strong indication of its versatility. Given that the swallowing impairments assessed are similar across different levels of healthcare, this broad scope of application suggests the potential generalization of the instrument to other contexts, such as primary care and home care, without the current sample restricting its applicability, although we recognize the importance of future studies that explore these realities more specifically.

It is important to acknowledge that the present study, whose main objective was to obtain initial validity evidence based on the content and response processes of DEGLUTO, did not include an in-depth analysis of its diagnostic accuracy, such as sensitivity, specificity, and predictive values. Future studies and subsequent publications will address this step, crucial for validating the instrument's ability to correctly identify cases of OD compared to gold standard methods.

## CONCLUSION

This study obtained initial evidence of the validity of DEGLUTO: Clinical Assessment Instrument of Swallowing in Adults, based on content and response processes. The application of the Delphi method allowed for a thorough analysis of the items, resulting in item exclusion and reformulation to ensure greater clarity, clinical relevance, and representativeness of the aspects evaluated. In the response process analysis stage, the results indicated good acceptability of the instrument among professionals, high agreement on most items, and an average application time compatible with clinical routine, reinforcing its feasibility and applicability in different care contexts.

The findings reinforce DEGLUTO as a standardized, reliable, and useful tool for the clinical assessment of swallowing. Future studies should further investigate other validity evidence, with a view to consolidating the instrument as a reference in SLH clinical practice.

## Appendix A DEGLUTO: Clinical Assessment Instrument of Swallowing in Adults

Jadson S. Dutra Alves, Leandro de A. Pernambuco, Hipólito Magalhães

NAME______________________________________________ DATE ______________

SEX______ AGE_______ DOB: ____________ OCCUPATION_____________________

ADDRESS_____________________________________ PHONE___________________

**Table:**

**- Medical History**	**Not suggestive (No)**	**Suggestive (Yes)**
1. History of aspiration pneumonia or recurrent pneumonia		
2. Prolonged intubation (48 or more hours) or current or previous tracheostomy		
**- Behavioral Aspects**		
3. Absentmindedness or fluctuating alertness		
4. Difficulty in managing oropharyngeal secretions		
**- Gross Motor Function**		
5. Inadequate postural control: trunk and neck instability		
6. Signs of fatigability.		
**- Oral Motor Test**		
7. Possible alterations in the anatomy and physiology of the mouth, pharynx, and larynx		
8. Presence of dysarthria		
9. Impaired orofacial muscle tone		
10. Presence of oral apraxia		
11. Changes in orofacial sensitivity		
12. Accumulation of saliva in the oral cavity		
13. Absent or inefficient voluntary cough and throat clearing		
**Observation during swallowing tests: 5 ml of IDDSI 4 food; a portion of IDDSI 7 food (when possible); 3 ml, 5 ml, and 10 ml of IDDSI 0 food.**		
14. Difficulty swallowing on command		
15. Presence of oral residue after swallowing		
16. Presence of swallowing-related cough and throat clearing		
17. Difficulty initiating the pharyngeal phase		
18. Reduction in hyolaryngeal movement		
19. Change in voice quality after swallowing		
20. Multiple swallows per bolus (3 or more)		
**Three variables summarized from the categories above.**		
Total number of suggestive items in the 20 variables of the 5 categories: _____		
Total number of suggestive items in Behavioral Aspects and Gross Motor Function:_____		
Total number of suggestive items in the results of the Oral Motor Test and Observations During the Swallowing Tests:_____		

## Appendix B Guidelines for Calibrating Evaluators

**Table:**

**MEDICAL HISTORY**			
**ITEMS**			**PROCEDURES**
1. History of aspiration pneumonia or recurrent pneumonia	No – Not suggestive		Ask: Have you ever had pneumonia due to food? Do you get pneumonia frequently?
			Have you noticed an increase in mucus in your mouth, throat, and/or lungs recently?
	Yes – Suggestive		
2. Prolonged intubation (48 or more hours) or current or previous tracheostomy	No – Not suggestive		Ask:
			- Did you need to be intubated (tube through your mouth) for two days or more to breathe better?
	Yes – Suggestive		- Did you ever need a tracheostomy (opening in your neck) to breathe better?
**BEHAVIORAL ASPECTS**			
**ITEMS**	**OUTCOMES**	**INTERPRETATION OF THE OUTCOMES**	**PROCEDURES**
3. Absentmindedness or fluctuating alertness	Alert/awake – not suggestive	Fully alert, able to participate	Observe throughout the interview and mark at the end.
	
	Reduced alertness or lethargy – suggestive	Patient needs stimulation to remain alert; stimuli can be verbal and/or tactile; feels sleepy, closes eyes, or has fluctuating attention.	
4. Difficulty in managing oropharyngeal secretions	Patient can control their secretions – not suggestive	Regularly controls secretion, throat clearing, and coughing.	Observe throughout the interview and mark at the end.
	
	Patient cannot control their secretions – suggestive	Wet voice, drooling, constant presence of oral and pharyngeal secretions, and stasis of oropharyngeal secretions.	
**GROSS MOTOR FUNCTION**			
**ITEMS**	**OUTCOMES**	**INTERPRETATION OF THE OUTCOMES**	**PROCEDURES**
5. Inadequate postural control: trunk and neck instability.	Proper postural control – not suggestive	The patient has normal movement either in bed or in a chair; transfers from one place to another; and uses bed controls (in bedridden patients).	Observe throughout the interview and mark at the end.
	
	Inadequate postural control – suggestive	Patients with motor impairments, spasms, etc.	
		Inability to move/transfer/sit independently; requires assistance to move, sit, or use bed controls.	
6. Signs of fatigability	Absence of signs of fatigability – not suggestive	The patient has good stamina and can complete all requested tasks.	Observe throughout the interview and mark at the end.
	Presence of signs of fatigability – suggestive		
		The patient reports fatigue, asks for a break, or refuses to complete the requested tasks.	
**ORAL MOTOR TEST RESULTS**			
**ITEMS**	**OUTCOMES**	**INTERPRETATION OF THE OUTCOMES**	**PROCEDURES**
7. Possible alterations in the anatomy and physiology of the mouth, pharynx, and larynx	Absence of alterations – not suggestive	No changes	Observe the face and soft tissues at rest and during directed movement (elevation, lowering, lateralization, protrusion, retraction of the orofacial muscles); observe hard tissues at rest.
			Request the sustained vowel /a/.
	Presence of alterations – suggestive	Facial asymmetry, altered voice quality, missing teeth that compromise chewing, altered mobility of oropharyngeal structures, and anatomical alterations	
8. Presence of dysarthria	No dysarthria – not suggestive	Intelligibility equal to or greater than 95%	Observe throughout the interview and mark at the end.
	
	Presence of dysarthria – suggestive	Speech intelligibility is minimally, moderately, or severely impaired; anarthria. No speech due to global aphasia or aphasia not amenable to assessment.	
9. Impaired orofacial muscle tone	Normal facial tone – not suggestive	Proper facial symmetry and strength	Observation at rest and in motion (consider the tests performed in item 7).
			Two-finger palpation of the face and lips.
	Facial weakness – suggestive	Drooping face and/or reduced strength of orofacial muscles	
10. Presence of oral apraxia.	No apraxia – not suggestive	Normal control in performing motor sequences of the lips and tongue	Observation of movement (consider the tests performed in item 7).
			Unable to perform the movements voluntarily.
	Oral apraxia – suggestive	Presence of signs of oral (orofacial) apraxia.	
11. Changes in orofacial sensitivity	Good orofacial sensitivity – not suggestive	Patient can feel touch in various parts of the face or mouth/tongue	Gently touch the quadrant with the spatula and ask the patient to indicate where it was touched:
	Altered orofacial sensitivity – suggestive		- upper right and left (forehead);
		Limited ability to feel touch in the face and/or mouth (even with food in the mouth, does not feel it)	- middle right and left (cheeks);
			- lower right and left (above the upper lip right/left and below the lower lip right/left);
			Touch inside the mouth: anterior third of the tongue, right and left, middle right and left, and longitudinal sulcus.
			Ask if the patient feels any difference to the touch on one side compared to the other.
12. Accumulation of saliva in the oral cavity	Oral cavity without saliva accumulation – not suggestive	No accumulated saliva is observed in the oral cavity.	Observe the oral cavity without commanding to swallow.
	
	Oral cavity with saliva accumulation – suggestive	Stagnant saliva is observed in the oral cavity, along with sialorrhea..	
	
13. Absent or inefficient voluntary cough and throat clearing	Voluntary cough and throat clearing present and strong – not suggestive	Able to perform a strong cough and/or throat clearing on command.	Ask the person being evaluated to cough and clear their throat vigorously..
	
	Cough and throat clearing absent or weak – suggestive	Presence of a weak cough or no cough on command. Weakness/inability to perform throat clearing on command.	
	
**OBSERVATION DURING SWALLOWING TESTS**			
**ITEMS**	**OUTCOMES**	**INTERPRETATION OF THE OUTCOMES**	**PROCEDURES**
14. Difficulty swallowing on command	Coordinates swallowing on command – not suggestive	Consensus of the expert committee	Observe for the presence of voluntary swallowing. Request voluntary swallowing and observe whether or not it is performed.

	Does not coordinate it – suggestive		
15. Presence of oral residue after swallowing	Absence of residue after food ingestion – not suggestive	Consensus of the expert committee	Oral inspection after offering food.

	Presence of residue after food ingestion – suggestive		
16. Presence of swallowing-related cough and throat clearing	Absence of cough and/or throat clearing after eating – not suggestive	Consensus of the expert committee	Observe during food offering

	Presence of cough and/or throat clearing after eating – suggestive		
17. Difficulty initiating the pharyngeal phase	Initiates hyolaryngeal movement without difficulty after preparing the bolus - not suggestive	Observe whether the patient can initiate displacement of the hyolaryngeal complex without difficulty after preparing the bolus.	During swallowing, observe whether the hyoid bone moves upwards and forwards, and the larynx rises and approaches the middle finger after the bolus has been prepared. The movement should begin without difficulty, such as hesitation or multiple unsuccessful attempts.

	Does not initiate or has difficulty initiating hyolaryngeal movement after preparing the bolus - suggestive		
18. Reduction in hyolaryngeal movement	Adequate elevation – not suggestive	Observe while performing the four-finger test:	During swallowing, observe whether the hyoid bone moves upwards and forwards, and the larynx rises and approaches the middle finger.
		Index finger under the submental triangle, middle finger touching the hyoid bone, ring finger over the thyroid cartilage, and little finger over the cricoid cartilage.	
	Reduced elevation – suggestive		
19. Change in voice quality after swallowing	Absence of alterations – not suggestive	Observe for any wet, tense, or strained voice… Any vocal changes after swallowing.	Ask the patient to pronounce the vowel /a/ after swallowing..

	Presence of alterations – suggestive		
20. Multiple swallows per bolus (3 or more)	Up to 2 swallows per bolus – not suggestive	Observe the number of swallows performed per offering.	Observe during food offering

	3 or more swallows per bolus – suggestive		
