# Supplementary material for: DEGLUTO: initial validity evidence of a clinical swallowing assessment tool for adults
Source: Codas. 2026 May 8;38(3):e20250153. doi: 10.1590/2317-1782/e20250153en (PMC13193195; doi:10.1590/2317-1782/e20250153en)
Supplement: Translation

# Translation: DEGLUTO: evidências de validade iniciais de um instrumento para avaliação clínica da deglutição em adultos

**DOI:** 10.1590/2317-1782/e20250153pt

**Authors:** Jadson Stheferson Dutra Alves, Leandro de Araújo Pernambuco, Hipólito Virgilio Magalhães

**Affiliations:** 1 Departamento de Fonoaudiologia, Universidade Federal do Rio Grande do Norte – UFRN - Natal (RN), Brasil.; 2 Departamento de Fonoaudiologia, Universidade Federal de Pernambuco – UFPE - Recife (PE), Brasil.

**Keywords:** Deglutição, Transtornos de Deglutição, Avaliação, Estudo de Validação, Fonoaudiologia

## Abstract

**Objetivo:**

desenvolver e verificar as evidências de validade de conteúdo do DEGLUTO, um instrumento de avaliação clínica da deglutição para pessoas adultas.

**Método:**

Estudo metodológico, do tipo transversal. A primeira fase validou o conteúdo pelo método Delphi com 13 juízes, analisando 28 itens e utilizando IVC, CVC e Kappa para concordância. A segunda fase avaliou processos de resposta com aplicação em 210 pacientes adultos e idosos por dez fonoaudiólogos, analisando tempo de aplicação, aceitabilidade e concordância interavaliadores.

**Resultados:**

Na validação de conteúdo, sete itens foram removidos e vinte e um reformulados, resultando em 21 itens com IVC médio > 0,90 e concordância substancial a quase perfeita. Nos processos de resposta, tempo médio de aplicação de 15 minutos, boa aceitabilidade e alta concordância na maioria dos itens foram observados. Um item foi excluído, consolidando a versão final com 20 itens.

**Conclusão:**

O DEGLUTO apresentou evidências iniciais robustas de validade, demonstrando aplicabilidade clínica, clareza e adequação teórica, qualificando-se como ferramenta promissora para avaliação da disfagia orofaríngea na prática assistencial.

## INTRODUÇÃO

A disfagia orofaríngea (DO) é uma condição clínica caracterizada pelo comprometimento funcional da deglutição, que dificulta ou impede a ingestão segura e eficiente de alimentos e líquidos, impactando negativamente a qualidade de vida dos indivíduos^(1)^. Está associada a complicações potencialmente graves, como desnutrição, desidratação, pneumonia aspirativa e aumento na taxa de mortalidade, o que reforça a necessidade de uma avaliação sistemática e precisa para seu diagnóstico e manejo adequados^(2)^.

A DO é uma condição comum que pode afetar uma ampla variedade de populações clínicas, com prevalência estimada em até 33,7% na população geral, sendo ainda mais elevada em indivíduos hospitalizados (41%) e em pessoas com diagnóstico de acidente vascular encefálico (55,2%)^(3)^.Entre idosos, a ocorrência varia de 18,3% em indivíduos da comunidade a 46,9% em residentes de instituições de longa permanência^(4)^. Em outras condições neurológicas, como a Doença de Parkinson, pode acometer até 80% dos pacientes^(5)^. Já em pessoas com câncer de cabeça e pescoço, sobretudo após intervenções como cirurgia e radioterapia, a disfagia afeta aproximadamente 50% dessa população^(6)^.

Diante desse cenário, a avaliação clínica da deglutição torna-se uma etapa essencial para o diagnóstico precoce e o manejo adequado da disfagia^(2)^. Nesse sentido, o uso de instrumentos submetidos ao processo de validação fortalece a prática baseada em evidências, proporcionando maior precisão diagnóstica e contribuindo para a definição de condutas terapêuticas mais seguras, eficazes e individualizadas^(4)^.

No entanto, para que esses instrumentos cumpram seu papel clínico de forma confiável, é imprescindível que sejam desenvolvidos e avaliados com base em critérios robustos de validade, a qual se refere à adequação das inferências feitas a partir dos resultados de um instrumento, sendo um atributo central para sua utilidade clínica^(7)^. De acordo com a ISPOR (The Professional Society for Health Economics and Outcomes Research), a construção da validade deve considerar o construto-alvo, a população de interesse, o contexto de aplicação e a consistência das interpretações resultantes do uso do instrumento^(7)^.

Nesse contexto, a validade de conteúdo representa uma das etapas fundamentais no desenvolvimento de instrumentos clínicos. Ela diz respeito ao grau em que os itens são representativos do construto-alvo e adequados ao contexto em que o instrumento será aplicado^(7)^. Segundo as boas práticas da ISPOR, essa análise deve ser conduzida por especialistas qualificados, que avaliem a clareza, a relevância e a abrangência dos itens, garantindo sua aderência à definição conceitual proposta e ao uso pretendido^(7-9)^.

Complementarmente, a ISPOR também destaca a importância de compreender como os respondentes interagem com o instrumento, assegurando que os itens sejam interpretados de maneira coerente com o construto-alvo^(9)^. Essa etapa, relacionada à análise dos processos de resposta, contribui para fortalecer a validade do instrumento, especialmente quando se trata de medidas baseadas em avaliação clínica, como os ClinROs. A observação e interpretação desses processos fornece evidências qualitativas essenciais para garantir que o instrumento produza resultados consistentes e úteis na prática clínica^(7,9)^.

Instrumentos validados são essenciais para diagnósticos seguros e condutas terapêuticas eficazes no manejo da disfagia. Para isso, devem ser construídos com rigor metodológico e aplicabilidade clínica. Segundo diretrizes do ISPOR, a validade de um instrumento considera tanto a representatividade dos itens quanto a forma como são interpretados pelos respondentes. Assim, este estudo teve como objetivo obter evidências iniciais de validade baseada no conteúdo e nos processos de resposta do DEGLUTO, conforme recomendações metodológicas para instrumentos ClinRO^(7-9)^.

## MÉTODO

### Desenho do estudo

Trata-se de um estudo metodológico voltado à obtenção de evidências iniciais de validade baseada no conteúdo e nos processos de resposta do DEGLUTO: Instrumento de Avaliação Clínica da Deglutição em Adultos (Apêndice A). O objetivo é subsidiar, em etapas futuras, a continuidade do processo de validação do instrumento em uma população de adultos e idosos, conforme as boas práticas metodológicas preconizadas pelo ISPOR – International Society for Pharmacoeconomics and Outcomes Research^(7-9)^.

O projeto foi encaminhado ao Comitê de Ética em Pesquisa do Hospital Universitário Onofre Lopes da Universidade Federal do Rio Grande do Norte (HUOL/UFRN), em consonância com a Resolução 466/2012, do Conselho Nacional de Saúde e aprovado sob número de parecer 6.934.805. Os fonoaudiólogos colaboradores que aceitaram participar da pesquisa, bem como os pacientes nos quais o instrumento foi aplicado, assinaram o Termo de Consentimento Livre e Esclarecido (TCLE).

### Obtenção de evidências de validade baseada no conteúdo

Neste estudo, foi empregada a técnica Delphi, método estruturado que visa alcançar consenso entre especialistas por meio de rodadas sucessivas de avaliação, realizadas de forma anônima e independente. Esse procedimento favorece decisões embasadas e reduz a influência de vieses grupais^(10)^. A cada rodada, os participantes recebiam um resumo estatístico das respostas anteriores, possibilitando a revisão e o refinamento de suas avaliações.

A versão brasileira do Northwestern Dysphagia Patient Check Sheet (NDPCS), previamente traduzida e adaptada transculturalmente, foi utilizada como base para a elaboração do conjunto inicial de 28 itens do DEGLUTO. Esses itens foram submetidos à avaliação dos juízes especialistas quanto à clareza, relevância e adequação ao construto proposto (identificação da disfagia orofaríngea).

### Local de pesquisa e período de referência

A etapa de obtenção de evidências de validade de conteúdo foi realizada de forma remota, com os juízes especialistas contatados por e-mail ao longo de três rodadas de avaliação. A coleta e análise dos dados ocorreram entre julho e novembro de 2024, período durante o qual os especialistas participaram ativamente do processo de revisão e refinamento do instrumento, conforme os princípios metodológicos previamente descritos.

### População

A amostra foi composta por juízes especialistas em disfagia orofaríngea, selecionados por conveniência, com base em sua experiência clínica, docente e/ou em pesquisa na área, com atuação mínima de cinco anos. A seleção foi realizada em reunião entre os pesquisadores responsáveis, considerando critérios de reconhecimento profissional e expertise no tema. Após essa definição, convites formais foram enviados por e-mail, totalizando 23 especialistas convidados para compor o painel de avaliação.

### Procedimentos e instrumentos de coleta de dados

Foram enviados aos participantes, por e-mail, uma carta-convite com orientações sobre a pesquisa, o Termo de Consentimento Livre e Esclarecido (TCLE) e a versão preliminar do instrumento a ser avaliado.

A avaliação foi conduzida por meio de formulário eletrônico estruturado com base em escala do tipo Likert: "concordo plenamente", "concordo", "neutro", "discordo" e "discordo fortemente". Cada item do DEGLUTO foi analisado quanto à sua relevância, atualidade, clareza e abrangência. Em casos de discordância, os juízes deveriam justificar suas respostas e sugerir alterações, além de poder incluir comentários ou propor novos itens julgados pertinentes.

O processo ocorreu em três rodadas sucessivas. Ao final de cada etapa, as respostas foram sistematicamente compiladas e discutidas em reuniões entre os pesquisadores, com base na literatura científica. Nesses encontros, as contribuições dos juízes foram avaliadas criteriosamente e utilizadas para a manutenção, modificação ou exclusão dos itens, de forma a assegurar a consistência conceitual e a adequação do conteúdo ao objetivo do instrumento.

### Análise dos dados

Os dados foram organizados em planilha eletrônica no formato .xlsx e analisados por meio do software Microsoft Excel 2024, utilizado também para a construção das tabelas descritivas.

A análise da validade de conteúdo do DEGLUTO baseou-se em três métricas complementares: o coeficiente Kappa, o Índice de Validade de Conteúdo (IVC) e o Coeficiente de Validade de Conteúdo (CVC). Adotou-se como critério mínimo para a manutenção ou exclusão dos itens o percentual de concordância igual ou superior a 70% entre os especialistas.

O coeficiente Kappa foi aplicado para verificar a consistência entre os avaliadores quanto às decisões sobre os itens. A interpretação seguiu a classificação proposta por Landis e Koch^(11)^: valores <0 indicam ausência de concordância; 0,00–0,19, concordância fraca; 0,20–0,39, regular; 0,40–0,59, moderada; 0,60–0,79, substancial; e 0,80–1,00, concordância quase perfeita.

O IVC foi calculado conforme os critérios de Pasquali^(12)^, sendo dividido em duas dimensões: o IVC por item (IVC-I), que corresponde à proporção de respostas positivas (“concordo” ou “concordo plenamente”), e o IVC geral (IVC-G), obtido pela média dos IVC-I por domínio. Consideraram-se aceitáveis valores superiores a 0,78 para o IVC-I e 0,90 para o IVC-G.

O CVC foi determinado com base na proposta de Hernández-Nieto^(13)^ em cinco etapas sucessivas. Inicialmente, obteve-se o CVC inicial (CVCi) a partir da média das pontuações dos avaliadores (escala de 1 a 5). Esse valor foi corrigido por um erro estimado (Pei), resultando no CVC corrigido (CVCc) para cada item. O CVC total (CVCt), por sua vez, correspondeu à média dos CVCc de cada dimensão do instrumento. Consideraram-se aceitáveis os valores superiores a 0,8013.

Os itens com índices abaixo dos parâmetros estabelecidos, bem como todas as sugestões qualitativas apresentadas pelos juízes, foram analisados pelos pesquisadores. Após discussão em reuniões de consenso, foram realizadas as modificações necessárias, resultando em uma versão preliminar revisada do instrumento.

### Obtenção de evidências de validade com base nos processos de resposta

Esta etapa foi conduzida com a participação de 10 fonoaudiólogos colaboradores, que aplicaram o DEGLUTO em diferentes contextos clínicos. Após a aplicação, os profissionais foram convidados a avaliar aspectos relacionados à clareza e abrangência dos itens, à compreensão dos pacientes durante a utilização do instrumento, bem como à aplicabilidade clínica geral.

Também foram considerados o tempo médio necessário para aplicação e a aceitabilidade do instrumento entre os avaliadores. A análise desses elementos possibilitou identificar como os respondentes interagiram com o DEGLUTO, garantindo que os itens fossem interpretados conforme o construto pretendido.

### Local de pesquisa e período de referência

A pesquisa ocorreu no ambulatório de disfagia orofaríngea do HUOL/UFRN, e nos locais de atuação dos fonoaudiólogos colaboradores. Os cenários de aplicação abrangeram dois hospitais públicos de alta complexidade (um hospital geral universitário e um hospital de referência em neurologia, trauma e queimaduras), um hospital privado de grande porte, e dois ambulatórios hospitalares públicos especializados no atendimento a pacientes com disfagia orofaríngea.

As aplicações foram realizadas em unidades de terapia intensiva (UTIs), enfermarias (clínicas e cirúrgicas), ambulatórios (disfagia, neurologia e cabeça e pescoço) e pronto-socorro. Esta ampla distribuição geográfica e tipológica dos ambientes de pesquisa teve como objetivo testar a aplicabilidade do instrumento em diferentes realidades assistenciais, desde o cuidado agudo até o acompanhamento ambulatorial. Todas as etapas do estudo seguiram os critérios éticos e metodológicos previamente aprovados pelo Comitê de Ética em Pesquisa. A coleta de dados foi realizada entre novembro de 2024 e fevereiro de 2025.

### População

A amostra do estudo foi composta por 210 pacientes adultos e idosos (média de 73,67 anos, 58,10% > 70 anos, maioria feminina) encaminhados para avaliação fonoaudiológica por queixas de disfagia orofaríngea. Selecionada por conveniência, incluiu uma vasta gama de condições clínicas associadas à disfagia, como idosos fragilizados, patologias neurológicas (Acidente Vascular Encefálico -AVE, doenças neurodegenerativas), agravos por internação prolongada, pós-intubação, traqueostomia, uso de vias alternativas de alimentação, doenças respiratórias descompensadas, câncer/queimaduras de cabeça e pescoço, e trauma. Essa diversidade visou assegurar a representatividade e a ampla aplicabilidade do DEGLUTO em diferentes contextos, conforme a recomendação de 10 pacientes/item.

A aplicação do DEGLUTO foi realizada por dez fonoaudiólogos colaboradores, todos com mais de cinco anos de formação e experiência no manejo da disfagia orofaríngea. Do total, quatro possuíam título de mestre, um de doutor, um com residência multiprofissional, um especialista stricto sensu e três atuavam exclusivamente na assistência clínica. Sete desses profissionais realizaram a aplicação do instrumento em hospitais. Os demais três colaboradores atuaram de forma ambulatorial. Essa diversidade profissional e institucional possibilitou uma análise abrangente da aplicabilidade clínica do DEGLUTO em diferentes cenários assistenciais.

### Procedimentos e instrumentos de coleta de dados

O processo envolveu a aplicação do DEGLUTO em diversos contextos clínicos pelos fonoaudiólogos colaboradores. Para assegurar uniformidade, todos os profissionais receberam orientações padronizadas em reunião presencial com o pesquisador, ocasião em que o manual de calibração do instrumento foi detalhado e entregue (Apêndice B).

Os fonoaudiólogos aplicaram o DEGLUTO em pacientes com queixas de disfagia orofaríngea, avaliando aspectos como clareza e abrangência dos itens, compreensão dos pacientes, aplicabilidade clínica do instrumento, tempo médio de aplicação e aceitabilidade geral.

Após o término das aplicações, cada profissional recebeu um formulário eletrônico individual, encaminhado por e-mail, no qual registrou suas percepções sobre a adequação do instrumento, possíveis dificuldades encontradas e sugestões de aprimoramento. Essas informações foram analisadas de forma sistemática, com o intuito de identificar aspectos a serem ajustados no instrumento, promovendo maior precisão e utilidade prática.

Cada item do DEGLUTO foi avaliado com base em duas escalas do tipo Likert, ambas com cinco opções de resposta: “concordo plenamente”, “concordo”, “neutro”, “discordo” e “discordo fortemente”. A primeira escala referia-se à adequação do item segundo a percepção do aplicador; a segunda, à clareza e compreensão demonstradas pelo paciente. Além das escalas, os formulários continham campos abertos para comentários qualitativos, permitindo uma análise mais aprofundada sobre a experiência de uso do instrumento.

### Análise dos dados

As análises das etapas de validade de conteúdo e de validade baseada nos processos de resposta foram conduzidas de forma padronizada, utilizando os mesmos critérios metodológicos e ferramentas estatísticas. Foram aplicados IVC, o CVC e o coeficiente Kappa, permitindo mensurar a concordância entre avaliadores, a representatividade dos itens e sua clareza no contexto clínico.

Essa uniformização metodológica garantiu maior consistência às análises e possibilitou a comparabilidade entre as evidências obtidas nas diferentes etapas do processo de validação. A utilização de múltiplos indicadores estatísticos também contribuiu para uma avaliação mais robusta da qualidade do instrumento, alinhando-se às recomendações das boas práticas internacionais para validação de instrumentos clínicos.

## RESULTADOS

### Obtenção de evidências de validade com base no conteúdo

Dos 23 juízes convidados para participar desta etapa, 13 aceitaram o convite e responderam à primeira rodada do questionário. Dos 13, 7 tinham atuação predominantemente acadêmica (professores universitários e pesquisadores com mais de 5 anos de experiência em disfagia e publicações relevantes na área) e 6 possuíam maior experiência na prática clínica e assistencial (fonoaudiólogos com mais de 5 anos de atuação em hospitais de alta complexidade e ambulatórios de disfagia). A diversidade de perfis entre os juízes buscou garantir uma avaliação abrangente tanto da perspectiva teórica quanto da aplicabilidade prática do instrumento

### Rodada I

A versão inicial do DEGLUTO continha 28 itens e, após a primeira rodada de avaliação, cinco questões foram removidas por apresentarem índice de concordância abaixo de 70% entre os juízes, baixa relevância clínica e pouca relação direta com o construto avaliado. Além disso, 21 itens passaram por reformulação, com ajustes na redação e estrutura para favorecer a clareza, reduzir ambiguidades e garantir maior precisão na aplicação. Essas modificações buscaram aprimorar a compreensão dos avaliadores, assegurando que os itens fossem interpretados de maneira uniforme e alinhados às necessidades da prática clínica. As questões excluídas foram as seguintes: 1. História de Pneumonia Recorrente, 7. Atenção/habilidade de interação, 9. Consciência das secreções, 14. Habilidade para seguir direções e 19. Contração faríngea no gag. Os índices de concordância globais nesta rodada foram Kappa = 0,70, IVC = 0,90 e CVC = 0,90, indicando uma concordância substancial entre os juízes e a adequação dos itens remanescentes.

### Rodada II

Na segunda rodada, 11 dos 13 juízes responderam ao questionário, resultando na ausência de dois especialistas, ambos com experiência clínica. Dessa forma, o grupo avaliador ficou composto por 7 juízes acadêmicos e 4 clínicos.

O instrumento revisado continha 23 questões restantes. Dessas, 3 apresentaram índice de concordância inferior a 70%. Sendo elas: 2. Apresenta episódios frequentes de febre, 6. Não cooperativo e 7. Falta de consciência do problema de deglutição. Após revisão da literatura e consenso dos pesquisadores responsáveis, as questões 2. Apresenta episódios frequentes de febre e 7. Falta de consciência do problema de deglutição foram removidas, enquanto a questão 6. Não cooperativo, foi reformulada para “comportamento inadequado durante as refeições”. As demais 21 questões foram mantidas, atingindo uma média de concordância de 85%. Os índices globais nesta rodada foram Kappa = 0,75, IVC = 0,92 e CVC = 0,91, demonstrando melhora na consistência do instrumento.

### Rodada III

Já na terceira rodada, 9 dos 11 juízes que participaram da fase anterior responderam ao questionário, sendo que um especialista acadêmico e um clínico não participaram dessa etapa, consolidando a banca final com 6 acadêmicos e 3 assistentes.

O foco exclusivo foi a reavaliação da questão reformulada 6. Comportamento inadequado durante as refeições, que obteve 70% de concordância entre os juízes. Os índices de concordância para este item foram Kappa = 0,22, IVC = 0,78 e CVC = 0,82, indicando um nível de concordância regular.

Essa etapa concluiu o processo de atualização do instrumento pelos juízes, seguindo os princípios do método Delphi, assegurando que as modificações realizadas estivessem coerentes com o objetivo do estudo e adequadas ao construto avaliado pelo instrumento.

Os resultados obtidos durante o processo de avaliação e atualização do instrumento estão apresentados na Tabela 1, organizados de forma a destacar as principais alterações realizadas ao longo do processo e os índices de concordância obtidos ao final das três rodadas.

**Tabela 1:** Obtenção de Evidências de Validade com Base no Conteúdo do DEGLUTO: Instrumento de Avaliação Clínica da Deglutição em Adultos

**nº**	**Item Original**	**Resposta dos Juízes**	**Atualização pelos Pesquisadores**	**Índice de Concordância Final (%)**
1	História de pneumonia recorrente	Item redundante; sugerem combinação com outro	Item removido	69%
2	Picos de temperatura frequentes	Pouca relação direta com a disfagia; sugerem remoção	Item removido	25%
3	Problema de pneumonia aspirativa	Concordância total com a relevância do item	Histórico de pneumonia aspirativa ou pneumonias de repetição	100%
4	Intubação de longa duração (1 semana ou +) ou traqueostomia (6 meses ou +)	Necessidade de melhor definição do tempo de intubação e traqueostomia	Intubação prolongada (48h ou +) e/ou presença de traqueostomia (atual ou prévia)	100%
5	Estado de alerta	Termo vago; sugerem especificar rebaixamento ou flutuação	Rebaixamento ou flutuação do estado de alerta	100%
6	Cooperação	Sugestão de detalhar comportamento durante refeições	Comportamento inadequado durante as refeições	70%
7	Atenção/habilidade de interação	Critério subjetivo; não relevante para disfagia	Item removido	67%
8	Consciência do problema de deglutição	Pouca aplicabilidade clínica; sugerem remoção	Item removido	62,50%
9	Consciência das secreções	Baixa confiabilidade clínica; ausência de consenso sobre relevância	Item removido	56,25%
10	Habilidade de manusear secreções	Concordam que a formulação do item deve ser mais clara	Dificuldade em manejar secreções orofaríngeas	90%
11	Controle postural	Item relevante; necessidade de especificar	Controle postural inadequado: instabilidade de tronco e pescoço	100%
12	Fatigabilidade	Confirmam relevância, mas recomendam maior objetividade	Sinais de fatigabilidade	90%
13	Anatomia/fisiologia oral, faríngea e laríngea	Necessidade de reformulação para maior aplicabilidade	Possíveis alterações na anatomia e fisiologia oral, faríngea ou laríngea	90%
14	Habilidade para seguir direções	Item não essencial para avaliação da disfagia	Item removido	56%
15	Disartria	Sugerem manter como critério relevante para disfagia	Presença de disartria	100%
16	Tonicidade orofacial	Concordam com a importância, mas recomendam ajuste na terminologia	Alteração do tônus orofacial	90%
17	Apraxia oral	Item relevante; necessário melhor detalhamento	Presença de apraxia oral	81%
18	Sensibilidade orofacial	Item relevante, com necessidade de ajustes na descrição	Alterações na sensibilidade orofacial	100%
19	Contração faríngea no reflexo do gag	Baixa correlação com disfagia; recomendam remoção	Item removido	69%
20	Deglutição de saliva	Necessário detalhar o critério de avaliação	Acúmulo de saliva em cavidade oral	100%
21	Tosse e pigarro voluntários	Item relevante, mas precisa de reformulação para melhor clareza	Ausência ou ineficiência de tosse e pigarro voluntários	100%
22	Apraxia da deglutição	Sugerem refinamento da terminologia para maior precisão	Dificuldade em realizar deglutição sob comando	90%
23	Resíduo oral	Concordam com a relevância; recomendam maior clareza	Presença de resíduo oral após a deglutição	100%
24	Tosse e pigarro	Importante para avaliação da disfagia; manter	Presença de tosse e pigarro relacionados à deglutição	100%
25	Atraso na deglutição faríngea	Sugerem maior detalhamento do atraso na fase faríngea	Dificuldade em iniciar a fase faríngea	81%
26	Redução na elevação laríngea	Ajuste necessário para maior especificidade na descrição	Redução no movimento hiolaríngeo	100%
27	Voz molhada	Concordância com a relevância clínica; ajustes na redação	Alteração na qualidade vocal após deglutição	100%
28	Deglutições múltiplas	Sugerem explicitação do critério numérico	Múltiplas deglutições por bolo alimentar (3 ou +)	100%

Após a definição das 21 questões preliminares do DEGLUTO, foi desenvolvido um manual de calibração para os aplicadores, com o objetivo de garantir a padronização na utilização do instrumento. Esse manual contém orientações detalhadas sobre a interpretação de cada item, critérios de pontuação e diretrizes para minimizar variações entre avaliadores, assegurando maior confiabilidade na coleta de dados. A padronização da aplicação é essencial para reduzir discrepâncias na avaliação e aprimorar a precisão dos resultados (Apêndice B).

### Obtenção de evidências de validade com base nos processos de resposta

A versão preliminar do DEGLUTO foi aplicada em um total de 210 pacientes por 10 fonoaudiólogos colaboradores.

A aplicação do instrumento revelou que os perfis de resposta variaram de forma consistente com a etiologia da disfagia nas diferentes populações clínicas estudadas. Observou-se que pacientes com afecções neurológicas, como AVE e doenças neurodegenerativas, apresentaram um maior número de itens sugestivos em todas as cinco categorias do instrumento. Os achados de maior destaque neste grupo incluíram alterações no teste motor oral, como disartria e alteração de tônus , e sinais de risco durante a deglutição, como tosse e alteração na qualidade vocal.

Em contraste, grupos cuja disfagia era secundária a um comprometimento sistêmico, como idosos frágeis, pacientes em pós-intubação, traqueostomia ou com internação prolongada, exibiram um padrão caracterizado predominantemente por instabilidade postural, fatigabilidade e ineficiência da tosse voluntária, refletindo a fraqueza muscular generalizada e o descondicionamento físico.

Adicionalmente, pacientes com câncer de cabeça e pescoço demonstraram um perfil mais concentrado em itens relacionados a alterações anatomofisiológicas , resultando em déficits funcionais específicos, como presença de resíduo oral e redução do movimento hiolaríngeo. Conforme esperado, indivíduos com histórico de pneumonia aspirativa ou com vias alternativas de alimentação pontuaram positivamente nos itens da história médica e apresentaram escores elevados nos demais domínios, corroborando a gravidade que justificou a intervenção inicial.

Os resultados demonstraram alta concordância para a maioria dos itens do instrumento, com valores de Kappa superiores a 0,80, indicando concordância substancial ou quase perfeita entre os aplicadores. Os índices de IVC e CVC também apresentaram valores acima de 0,90 para 20 dos 21 itens, reforçando a adequação do instrumento na prática clínica.

Apenas o item 4, “Comportamento inadequado durante as refeições”, apresentou resultados abaixo dos critérios estabelecidos para aceitação, com Kappa = 0,14, IVC = 0,75 e CVC = 0,82. Esses valores indicam baixa padronização na interpretação do item pelos avaliadores, o que motivou sua exclusão após análise crítica e revisão da literatura. A concordância entre os avaliadores está descrita na Tabela 2.

**Tabela 2:** Concordância entre os avaliadores para cada questão

**Dimensão**	**Item**	**Kappa**	**IVC**	**CVC**
**História médica**	1. Histórico de pneumonia aspirativa ou pneumonias de repetição.	1,00	1,00	1,00
	2. Intubação de longa duração (48h ou +) ou traqueostomia (atual ou prévia).	1,00	1,00	0,95
**Aspectos comportamentais**	3. Rebaixamento ou flutuação do estado de alerta.	1,00	0,71	0,92
	4. Comportamento inadequado durante as refeições.	0,14	0,75	0,82
	5. Dificuldade em manejar secreções orofaríngeas.	1,00	1,00	0,90
**Função motora ampla**	6. Controle postural inadequado: instabilidade de tronco e pescoço.	1,00	1,00	0,90
	7. Sinais de fatigabilidade.	1,00	1,00	0,92
**Teste motor oral**	8. Possíveis alterações na anatomia e fisiologia oral, faríngea e laríngea.	1,00	1,00	0,87
	9. Presença de disartria.	1,00	1,00	0,92
	10. Alteração do tônus orofacial.	1,00	1,00	0,95
	11. Presença de apraxia oral.	1,00	1,00	0,92
	12. Alterações na sensibilidade orofacial.	1,00	1,00	0,92
	13. Acúmulo de saliva em cavidade oral.	1,00	1,00	0,97
	14. Ausência ou ineficiência da tosse e pigarro voluntário.	1,00	1,00	0,95
**Observação durante as provas de deglutição**	15. Dificuldade em realizar deglutição sob comando.	1,00	1,00	0,95
	16. Presença de resíduo oral após a deglutição.	1,00	1,00	0,95
	17. Presença de tosse e pigarro relacionados à deglutição.	1,00	1,00	0,97
	18. Dificuldade em iniciar a fase faríngea.	1,00	1,00	0,95
	19. Redução no movimento hiolaríngeo.	1,00	1,00	0,95
	20. Alteração na qualidade vocal após deglutição.	1,00	1,00	0,95
	21. Múltiplas deglutições por bolo alimentar (3 ou +).	1,00	1,00	0,92
	Global	0,96	0,99	0,93

**Legenda:** IVC = Índice de Validação de Conteúdo; CVC = Coeficiente de Validação de Conteúdo. Fonte: autoria própria

A mensuração do tempo de aplicação revelou que a administração do DEGLUTO variou entre 12 e 20 minutos, com uma média de 15 minutos por paciente. A aceitabilidade do DEGLUTO foi avaliada por meio de questionário eletrônico. A análise das respostas indicou boa aceitação, com destaque para a objetividade, facilidade de aplicação e adequação do instrumento a diferentes contextos clínicos.

## DISCUSSÃO

A obtenção de evidências de validade com base no conteúdo e nos processos de resposta é fundamental para garantir que um instrumento reflita com precisão o construto avaliado e tenha aplicabilidade clínica. Esse processo segue as boas práticas internacionais da *ISPOR*, que orientam o desenvolvimento de instrumentos válidos, padronizados e relevantes para a prática^(7-9)^. O método Delphi permitiu uma análise criteriosa do DEGLUTO, resultando em ajustes que aprimoraram sua clareza e relevância clínica.

A exclusão do item “história de pneumonia recorrente” foi embasada na literatura, que aponta a complexidade da relação entre pneumonia e disfagia. Embora a aspiração seja um fator de risco, pneumonias recorrentes envolvem múltiplas variáveis, como imunidade, higiene oral, dependência funcional e comorbidades^(14)^. Sua inclusão, apesar de parecer aumentar a abrangência do instrumento, poderia gerar interpretações equivocadas e reduzir a especificidade do DEGLUTO, pois não necessariamente indicaria um problema de deglutição em si, mas uma consequência multifatorial.

A questão “atenção/habilidade de interação” foi excluída por ser um critério subjetivo e pouco específico para avaliação da disfagia. Embora déficits cognitivos possam interferir na alimentação, nem sempre determinam a presença de alterações na deglutição. Estudos indicam que a capacidade atencional pode influenciar o comportamento alimentar, mas sua relação direta com a eficácia da deglutição não é conclusiva^(15)^.

A questão “consciência das secreções” foi removida do instrumento devido à sua baixa confiabilidade clínica, uma vez que a percepção do paciente sobre a presença e manejo de secreções orais e faríngeas pode ser altamente variável. Essa variabilidade é ainda mais acentuada em indivíduos com comprometimento neurológico, que frequentemente apresentam redução da sensibilidade laríngea e faríngea, impactando sua capacidade de detectar e eliminar secreções acumuladas na via aérea superior^(14)^.

A exclusão da questão “habilidade para seguir direções" foi fundamentada no fato de que déficits cognitivos não necessariamente impactam diretamente a mecânica da deglutição. Pacientes podem apresentar dificuldades de compreensão sem comprometimento significativo na fase oral ou faríngea da deglutição, tornando essa questão menos relevante dentro do escopo do instrumento^(15)^.

A questão “contração faríngea” no reflexo do “gag” foi excluída por não apresentar correlação direta com a eficácia da deglutição. Estudos demonstram que a presença ou ausência desse reflexo não prediz o risco de aspiração e não constitui um marcador confiável para disfagia orofaríngea^(16)^. Indivíduos saudáveis podem não apresentar o reflexo sem prejuízo funcional, e sua ausência também não se associa a maiores taxas de aspiração em pacientes neurológicos^(16)^.

A questão “presença de episódios frequentes de febre” foi removida por sua baixa especificidade como indicador isolado de disfagia, uma vez que a febre é um sinal clínico inespecífico, frequentemente associado a diversas causas infecciosas não relacionadas à deglutição, como infecções urinárias, virais ou sepse. Embora possa ocorrer em casos de pneumonia aspirativa, nem sempre está presente, o que limita sua utilidade como marcador clínico e pode levar a interpretações equivocadas^(14)^. Sua inclusão poderia gerar vieses diagnósticos e levar a interpretações equivocadas.

A questão “consciência do problema de deglutição” foi excluída devido à baixa confiabilidade da autopercepção como critério diagnóstico. Em condições neurológicas, os pacientes frequentemente subestimam suas dificuldades de deglutição, o que pode resultar em falso senso de segurança e maior risco de aspiração^(4)^. Estudos com VED e VFD mostram que muitos não reconhecem a própria disfagia, especialmente na presença de aspiração silenciosa, o que compromete a acurácia diagnóstica ao confiar na percepção subjetiva^(5)^. Ao remover este item, o DEGLUTO foca em sinais objetivos e observáveis da disfagia, garantindo maior precisão e padronização na avaliação clínica.

A questão “cooperação” foi reformulada para “comportamento inadequado durante as refeições" para ampliar a aplicabilidade clínica e reduzir vieses. A nova redação foca em comportamentos observáveis (recusa, distração, resistência ao manejo), que impactam risco de aspiração, eficácia da alimentação e adesão, especialmente em populações neurológicas e psiquiátricas. Em demência avançada, apatia e resistência à alimentação assistida aumentam riscos nutricionais e respiratórios^(5,14)^. A reformulação alinha a avaliação à prática clínica e favorece intervenções eficazes.

O processo de validação de conteúdo do DEGLUTO resultou em 21 itens preliminares, com base em critérios metodológicos rigorosos que garantiram clareza, relevância e aplicabilidade clínica. As exclusões e reformulações foram fundamentadas na literatura e na experiência dos especialistas, priorizando marcadores clínicos objetivos e diretamente relacionados à DO. Esse refinamento contribuiu para uma avaliação mais padronizada e assertiva, favorecendo a identificação precoce de alterações na deglutição e maior segurança nas decisões clínicas em contextos hospitalares e ambulatoriais.

Após a etapa de validação de conteúdo, a análise dos processos de resposta foi fundamental para avaliar a clareza, aplicabilidade e compreensão dos itens por profissionais e pacientes, assegurando o uso consistente do instrumento na prática clínica^(17)^. A aplicação do DEGLUTO em diferentes cenários assistenciais, por fonoaudiólogos experientes, evidenciou sua funcionalidade e alta concordância entre os avaliadores na maioria dos itens, reforçando sua confiabilidade, padronização e aplicabilidade clínica.

A aplicação do DEGLUTO demonstrou que os perfis de resposta dos pacientes variaram consistentemente com a etiologia da disfagia, o que corrobora a literatura existente sobre a complexidade dessas alterações^(1,18)^. Pacientes neurológicos apresentaram mais achados sugestivos em todas as categorias, destacando déficits motores orais (disartria e alteração de tônus) e riscos durante a deglutição (tosse e alteração vocal), alinhado ao que é descrito sobre o impacto neurológico na deglutição^(5)^.

Em contraste, idosos frágeis e pacientes em pós-intubação, traqueostomia ou internação prolongada exibiram padrões de instabilidade postural, fatigabilidade e ineficiência da tosse, refletindo fraqueza muscular generalizada^(4)^. Já em pacientes com câncer de cabeça e pescoço, predominou a identificação de alterações anatomofisiológicas, como resíduo oral e redução do movimento hiolaríngeo, justificadas pelo comprometimento estrutural^(6)^. Por fim, o registro de histórico de pneumonia aspirativa e uso de vias alternativas de alimentação validou a capacidade do instrumento em identificar a gravidade preexistente da disfagia^(19)^.

Um ponto crítico identificado foi a baixa concordância no item “Comportamento inadequado durante as refeições”, com Kappa = 0,14, IVC = 0,75 e CVC = 0,82, abaixo dos critérios de aceitação. A inconsistência na interpretação pelos aplicadores comprometeu sua utilidade clínica, possivelmente devido à subjetividade envolvida em aspectos comportamentais, influenciados por fatores como cognição, motivação e estado emocional^(14)^. Sua exclusão visou preservar a objetividade e a reprodutibilidade do instrumento, evitando viés na avaliação e fortalecendo a precisão diagnóstica do DEGLUTO.

A análise do tempo de aplicação do DEGLUTO (média de 15 minutos, variando entre 12 e 20) foi considerada adequada para a prática clínica, alinhada à literatura sobre a viabilidade de instrumentos em contextos com restrições de tempo^(20)^. A variação foi influenciada pela gravidade do paciente, experiência do aplicador e familiaridade com o instrumento, conforme estudos de aplicabilidade clínica em disfagia^(20)^. Profissionais experientes tendem a reduzir o tempo, enquanto pacientes mais comprometidos exigem mais.

A aceitabilidade do DEGLUTO pelos profissionais foi positiva, sendo descrito como claro, sistematizado e de fácil aplicação. A literatura destaca que instrumentos com base científica e estrutura objetiva favorecem a adesão clínica, ao minimizar ambiguidades e variações interpretativas^(20)^.

A análise dos processos de resposta evidenciou que o DEGLUTO é viável, aplicável e confiável para a prática clínica. A alta concordância entre avaliadores, o tempo de aplicação adequado e os relatos positivos dos profissionais reforçam sua utilidade na avaliação da deglutição, contribuindo para diagnósticos mais precisos e condutas clínicas padronizadas.

Apesar dos resultados positivos, algumas limitações devem ser consideradas. Na etapa de validação de conteúdo, observou-se queda na participação dos juízes ao longo das rodadas do método Delphi. Porém, apesar da variação no número de participantes entre as rodadas, as decisões foram tomadas com base em critérios de concordância estatísticos rigorosos, que asseguram a validade do conteúdo mesmo com a flutuação do painel. Além disso, as reformulações e exclusões de itens foram sempre embasadas em discussão aprofundada entre os pesquisadores e na literatura científica atualizada, minimizando o impacto da menor adesão e garantindo a consistência conceitual do DEGLUTO.

Apesar da amostra, na etapa de processos de resposta, ter sido selecionada por conveniência e em contextos clínicos específicos, o processo seguiu diretrizes internacionalmente reconhecidas pela ISPOR. A diversidade dos cenários de aplicação do DEGLUTO neste estudo é um forte indicativo de sua versatilidade. Dadas as alterações de deglutição avaliadas serem semelhantes em diferentes níveis de atenção à saúde, essa abrangência de aplicação sugere a potencial generalização do instrumento para outros contextos, como atenção primária e cuidados domiciliares, sem que a amostra atual restrinja sua aplicabilidade, embora reconheçamos a importância de estudos futuros que explorem essas realidades de forma mais específica.

É importante reconhecer que o presente estudo, por ter como objetivo principal a obtenção de evidências iniciais de validade baseadas no conteúdo e nos processos de resposta do DEGLUTO, não incluiu uma análise aprofundada de sua acurácia diagnóstica, como sensibilidade, especificidade e valores preditivos. Essa etapa, crucial para validar a capacidade do instrumento em identificar corretamente casos de DO em comparação com métodos padrão ouro, será abordada em estudos futuros e publicações subsequentes.

## CONCLUSÃO

O presente estudo obteve evidências iniciais de validade do DEGLUTO: Instrumento de Avaliação Clínica da Deglutição em Adultos – com base no conteúdo e nos processos de resposta. A aplicação do método Delphi possibilitou uma análise criteriosa dos itens, resultando na exclusão e reformulação de questões para garantir maior clareza, relevância clínica e representatividade dos aspectos avaliados. Na etapa de análise dos processos de resposta, os resultados indicaram boa aceitabilidade do instrumento entre os profissionais, alta concordância na maioria dos itens e tempo médio de aplicação compatível com a rotina clínica, reforçando sua viabilidade e aplicabilidade em diferentes contextos assistenciais.

Os achados reforçam o DEGLUTO como uma ferramenta padronizada, confiável e útil para a avaliação clínica da deglutição. Estudos futuros deverão aprofundar a investigação de outras evidências de validade, com vistas à consolidação do instrumento como referência na prática clínica fonoaudiológica.

## Apêndice A DEGLUTO: Instrumento de Avaliação Clínica da Deglutição em Adultos

Jadson S. Dutra Alves, Leandro de A. Pernambuco, Hipólito Magalhães

NOME______________________________________________ DATA ______________

SEXO______ IDADE_______ DN: ____________ PROFISSÃO_____________________

ENDEREÇO_____________________________________ TELEFONE_______________

**Table:**

**- História Médica**	**Não sugestivo (Não)**	**Sugestivo (Sim)**
1. Histórico de pneumonia aspirativa ou pneumonias de repetição		
2. Intubação de longa duração ( 48h ou +) ou traqueostomia (atual ou prévia)		
**- Aspectos Comportamentais**		
3. Rebaixamento ou flutuação do estado de alerta		
4. Dificuldade em manejar secreções orofaríngeas		
**- Função Motora Ampla**		
5. Controle postural inadequado: instabilidade de tronco e pescoço		
6. Sinais de fatigabilidade		
**- Teste Motor Oral**		
7. Possíveis alterações na anatomia e fisiologia oral, faríngea e laríngea		
8. Presença de disartria		
9. Alteração do tônus orofacial		
10. Presença de apraxia oral		
11. Alterações na sensibilidade orofacial		
12. Acúmulo de saliva em cavidade oral		
13. Ausência ou ineficiência de Tosse e pigarro voluntários		
**Observação durante as provas de deglutição: 5ml de alimento IDDSI 4; uma porção de de alimento IDDSI 7 (quando possível); 3 ml, 5 ml e 10 ml de de alimento IDDSI 0.**		
14. Dificuldade em realizar deglutição sob comando		
15. Presença de resíduo oral após a deglutição		
16. Presença de tosse e pigarro relacionados à deglutição		
17. Dificuldade em iniciar a fase faríngea		
18. Redução no movimento hiolaríngeo		
19. Alteração na qualidade vocal após deglutição		
20. Múltiplas deglutições por bolo alimentar (3 ou +)		
**Três variáveis resumidas das categorias acima**		
Total de itens sugestivos nas 20 variáveis das 5 categorias: _____		
Total de itens sugestivos nos Aspectos Comportamentais e Função Motora		
Ampla:_____		
Total de itens sugestivos nos resultados do Teste Motor Oral e Observações Durante as Provas de Deglutição:_____		

## Apêndice B Roteiro para Calibração dos Avaliadores

**Table:**

**HISTÓRIA MÉDICA**			
**ITENS**			**CONDUTAS**
1.Histórico de pneumonia aspirativa ou pneumonias de repetição	Não – Não sugestivo		Perguntar: Você tem ou já teve pneumonia por causa da alimentação? Você tem pneumonia com frequência?
	Sim - Sugestivo		
			Notou aumento de secreção na boca, garganta e/ou pulmões recentemente?
2. Intubação de longa duração ( 48h ou +) ou traqueostomia (atual ou prévia)	Não – Não sugestivo		Perguntar:
	Sim – Sugestivo		- Você precisou ser entubado (tubo pela boca) por dois dias ou mais para respirar melhor?
			- Você precisou ser traqueostomizado (abertura no pescoço) alguma vez para respirar melhor?
**ASPECTOS COMPORTAMENTAIS**			
**ITENS**	**DESFECHOS**	**INTERPRETAÇÃO DOS DESFECHOS**	**CONDUTAS**
3. Rebaixamento ou flutuação do estado de alerta	Alerta/acordado – não sugestivo	Completamente alerta, hábil para participar	Observar durante toda a entrevista e marcar ao final
	Estado de alerta reduzido ou letárgico – sugestivo	Paciente precisa de estimulação para se manter alerta/esperto; estímulos podem ser verbal e ou tátil; sente-se com sono, fecha os olhos ou tem flutuação na atenção	
4.Dificuldade em manejar secreções orofaríngeas	Paciente consegue controlar suas secreções – não sugestivo	Regularmente controla a secreção, pigarreia, tosse.	Observar durante toda a entrevista e marcar ao final
		Voz molhada, sialorreia, presença de secreções orais e faríngeas constantes, estase de secreções orofaríngeas.	
	Paciente não consegue controlar suas secreções – sugestivo		
**FUNÇÃO MOTORA AMPLA**			
**ITENS**	**DESFECHOS**	**INTERPRETAÇÃO DOS DESFECHOS**	**CONDUTAS**
5. Controle postural inadequado: instabilidade de tronco e pescoço	Controle postural adequado – não sugestivo	Paciente tem movimento normal ou na cama ou na cadeira; transfere-se de um lugar para o outro; usa os controles da cama (nos acamados).	Observar durante toda a entrevista e marcar ao final
	Controle postural inadequado – sugestivo		
		Pacientes com comprometimentos motores, espasmos etc.	
		Inabilidade para se mover/transferir/sentar independentemente; necessita de cuidados para se mover, sentar-se ou utilizar os controles da cama	
6. Sinais de fatigabilidade	Ausência de sinais de fatigabilidade – não sugestivo	Paciente tem boa resistência, pode completar todos os comandos solicitados.	Observar durante toda a entrevista e marcar ao final
	Presença de sinais de fatigabilidade – sugestivo		
		Paciente refere cansaço, pede pausa ou recusa completar as tarefas solicitadas.	
**RESULTADOS DO TESTE MOTOR ORAL**			
**ITENS**	**DESFECHOS**	**INTERPRETAÇÃO DOS DESFECHOS**	**CONDUTAS**
7. Possíveis alterações na anatomia e fisiologia oral, faríngea e laríngea	Ausência de alterações – não sugestivo	Sem alterações	Observar face e estruturas moles em repouso e movimento dirigido (elevação, abaixamento, lateralização, protrusão, retração da musculatura orofacial); observar estruturas duras em repouso
		Assimetria facial, alteração na qualidade vocal, ausências dentárias que comprometam a mastigação, alteração mobilidade das estruturas orofaríngeas, alterações anatômicas	
	Presença de alterações – sugestivo		
			Solicitar vogal sustentada /a/
8.Presença de disartria	Sem disartria – não sugestivo	Inteligibilidade igual ou maior que 95%	Observar durante toda a entrevista e marcar ao final
	Presença de Disartria – sugestivo	Inteligibilidade de fala comprometida mínima, média ou severamente; anartria. Sem fala em decorrência de afasia global ou não passível de avaliação	
9. Alteração do tônus orofacial	Tônus facial normal – não sugestivo	Adequação na simetria e resistência facial	Observação em repouso e em movimento (considerar as provas realizadas no item 7).
	Fraqueza facial – sugestivo	Face caída e/ou redução na resistência de musculatura orofacial	Palpação bidigital da face e lábios
10. Presença de apraxia oral	Sem apraxia – não sugestivo	Controle normal na realização das sequências motoras de lábios e língua	Observação no movimento (considerar as provas realizadas no item 7).
	Apraxia oral – sugestivo	Sinais presentes de apraxia oral (orofacial)	Não consegue realizar os movimentos de forma voluntária
11. Alterações na sensibilidade orofacial	Boa sensibilidade orofacial – não sugestivo	Paciente hábil para sentir o toque em várias partes da face ou na boca/língua	Tocar com a espátula levemente no quadrante e pedir para o paciente sinalizar onde foi tocado:
	Alteração na sensibilidade orofacial – sugestivo	Demonstra habilidade limitada para sentir o toque na face e/ou na boca (mesmo com comida na boca, não a sente)	
			- superior direito e esquerdo (testa);
			- médio direito e esquerdo (faces);
			- inferior direito e esquerdo (acima do lábio superior D/E e abaixo do lábio inferior D/E);
			Tocar dentro da boca terço anterior da língua D e E, médio D e E e sulco longitudinal
			Perguntar se o paciente sente diferenças ao toque de um lado em relação ao outro
12. Acúmulo de saliva em cavidade oral	cavidade oral sem acúmulo de saliva – não sugestivo	Não observa-se a presença de saliva acumulada em cavidade oral.	Observar a cavidade oral, sem direcionar a deglutição.
	cavidade oral com acúmulo de saliva – sugestivo	Observa-se presença de estase de saliva em cavidade oral, presença de sialorreia.	
13. Ausência ou ineficiência de Tosse e pigarro voluntários	Tosse e pigarro voluntários presentes e fortes – não sugestivo	Hábil para realizar tosse forte e/ou pigarro sob comando.	Solicitar tosse e pigarro fortes ao avaliado.
		Presença de tosse fraca ou sem tosse sob comando. Fraqueza/inabilidade para fazer pigarro sob comando.	
	Tosse e pigarro ausente ou fracos – sugestivo		
**OBSERVAÇÃO DURANTE AS PROVAS DE DEGLUTIÇÃO**			
**ITENS**	**DESFECHOS**	**INTERPRETAÇÃO DOS DESFECHOS**	**CONDUTAS**
14. Dificuldade em realizar deglutição sob comando.	Coordena a deglutição sob comando – não sugestivo	Consenso do comitê de especialistas (CCE) .	Observar presença de deglutição voluntária. Solicitar deglutição voluntária e observar se há execução ou não.
	Não coordena - sugestivo		
15. Presença de resíduo oral após a deglutição	Ausência de resíduos após a ingestão do alimento – não sugestivo	Consenso do comitê de especialistas (CCE)	Inspeção oral após oferta de alimento
	Presença de resíduos após a ingestão do alimento- sugestivo		
16. Presença de tosse e pigarro relacionados à deglutição	Ausência de tosse e/ou pigarro após a ingestão alimentar – não sugestivo	CCE	Observar durante a oferta de alimento
	Presença de tosse e/ou pigarro após a ingestão alimentar - sugestivo		
17. Dificuldade em iniciar a fase faríngea	Inicia o movimento hiolaringeo sem dificuldade após preparar o bolo alimentar - não sugestivo	Observar se o paciente consegue iniciar o deslocamento do conjunto hiolaringeo sem dificuldades após preparar o bolo alimentar.	Durante a deglutição, observar se, após finalizada a preparação do bolo alimentar, o hioide se desloca para cima e anteriormente e a laringe se eleva e se aproxima do dedo médio. Espera-se que o movimento inicie sem dificuldades como, por exemplo, hesitações ou múltiplas tentativas sem êxito.
	Não inicia ou tem dificuldades para iniciar o movimento hiolaringeo após preparar o bolo alimentar - sugestivo		
18. Redução no movimento hiolaríngeo	Elevação adequada – não sugestivo	Observar realizando a prova dos 4 dedos:	Durante a deglutição, observar se o hioide se desloca para cima e anteriormente e a laringe se eleva e se aproxima do dedo médio.
	Elevação reduzida - sugestivo	Indicador sob a região do trígono submentual, o dedo médio tocando o osso hióide, **anelar sobre a cartilagem tireoidea e mínimo sobre a cricoidea.**	
19. Alteração na qualidade vocal após deglutição	Ausência de alterações – não sugestivo	observar presença de voz molhada, tensa, estrangulada… Qualquer alteração vocal após a deglutição.	Pedir para o paciente emitir a vogal /a/ após a deglutição.
	Presença de alterações - sugestivo		
20. Múltiplas deglutições por bolo alimentar (3 ou +)	Presença de até 2 deglutições por bolo – não sugestivo	Observar o número de deglutições realizadas por oferta	Observar durante a oferta de alimento
	Presença de 3 ou mais deglutições por bolo - sugestivo
